# Endoscopic mucosal resection for Barrett’s neoplasia: Long-term outcomes from the largest Canadian single-center experience

**DOI:** 10.1055/a-2602-8961

**Published:** 2025-06-17

**Authors:** Yusuke Fujiyoshi, Kareem Khalaf, Daniel Tham, Mary Raina Angeli Fujiyoshi, Catherine J. Streutker, Natalia C. Calo, Jeffrey D. Mosko, Gary R. May, Norman E. Marcon, Christopher W. Teshima

**Affiliations:** 1 10071 Division of Gastroenterology, St. Michael's Hospital, University of Toronto, Toronto, ON, Canada; 2 6363 Department of Medicine, Division of Gastroenterology, University of Ottawa, Ottowa Hospital Research Institute, Ottawa, ON, Canada; 3 10071 Department of Laboratory Medicine, St. Michael's Hospital, University of Toronto, Toronto, ON, Canada

**Keywords:** Endoscopy Upper GI Tract, Barrett's and adenocarcinoma, Endoscopic resection (ESD, EMRc, ...), RFA and ablative methods

## Abstract

**Background and study aims:**

Endoscopic mucosal resection (EMR) remains an important treatment for high-grade dysplasia (HGD) and early esophageal adenocarcinoma (EAC) in Barrett’s esophagus (BE). However, there are limited data regarding long-term recurrence rates. This study aimed to investigate the neoplasia recurrence rate following EMR with long-term follow-up.

**Methods:**

This was a retrospective cohort study at a tertiary-referral center in Canada. Patients with Barrett’s neoplasia (HGD/EAC) treated with EMR between January 2001 and December 2023 were included. The primary outcome was long-term neoplasia recurrence rate after complete remission of neoplasia (CRN). Secondary outcomes were residual/metachronous neoplasia rate at first follow-up, CRN rate, and long-term rate of patients successfully managed by endoscopy.

**Results:**

A total of 552 patients (83.7% male, mean age 66.3 years) were included (HGD: 22.5%, EAC: 77.5%). After EMR, 475 patients were deemed to have had successful endoscopic resection (low lymph-node metastasis risk with tumor-free deep margin), 455 of whom underwent surveillance follow-up. At first follow-up, residual/metachronous neoplasia was observed in 20.9% (95/455), but 95.6% (435/455) eventually achieved CRN after undergoing a median of two EMR sessions (interquartile range: 1–4). As a primary outcome, the 5-year neoplasia recurrence rate was 10.5%, the 10-year rate was 21.6%, and the 15-year rate was 34.9%. During surveillance, neoplasia recurrence was observed in 38 patients, but 68.4% of them (26/38) were managed with endoscopic therapy. The overall rate of patients successfully managed by endoscopy was 93.0% (423/455).

**Conclusions:**

While the success rate of EMR for BE is excellent, this study highlights substantial long-term risk of neoplastic recurrence, underscoring the need for indefinite surveillance for patients who had HGD or EAC.

## Graphical abstract

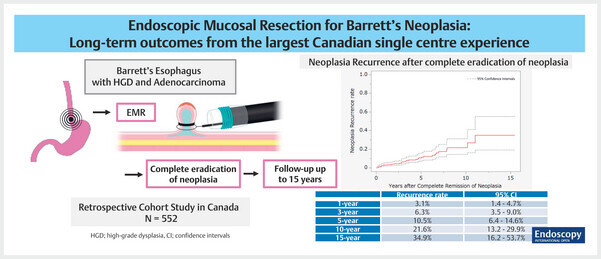

## Introduction

Barrett's esophagus (BE) is deﬁned by replacement of the normal squamous epithelium of the distal esophagus with metaplastic intestinal-type columnar epithelium. This condition is associated with development of dysplasia and esophageal adenocarcinoma (EAC) 1 . Endoscopic mucosal resection (EMR) has been one of the main endoscopic approaches for managing BE, especially in cases involving nodular high-grade dysplasia (HGD) and early-stage EAC 2 3 . EMR has been shown to have high therapeutic efficacy and low complication rates and has long been the cornerstone of management of Barrett's neoplasia 4 5 6 .

Current guidelines recommend endoscopic resection of visible nodular lesions in BE, both for treatment as well as for histopathological staging 7 8 9 . After EMR, patients are categorized into high- or low-risk groups according to risk for lymph node metastasis and local recurrence, based on histopathological findings 9 . Low-risk patients are considered to have had successful endoscopic resection and additional ablation and ongoing surveillance with endoscopy is recommended for them, whereas high-risk patients are advised to undergo esophagectomy. For patients undergoing surveillance following successful endoscopic resection, identification of recurrent neoplasia is critical 10 . Also, given that Barrett’s HGD and early-stage EAC are typically not immediately life-threatening, long-term follow-up is vital.

However, there are relatively few studies that have examined very long-term recurrence rates following EMR 3 . Guidelines recommend performing surveillance endoscopy for 10 years 9 , but there is no research providing data that extend beyond a 10-year period. Therefore, this study aimed to investigate the long-term recurrence rate of BE neoplasia following successful endoscopic resection by EMR.

## Patients and methods

### Study design and setting

A retrospective cohort study was conducted at a single tertiary referral center in Toronto, Canada, utilizing a prospectively maintained database of patients diagnosed with BE. The study covered the period from January 2001 to December 2023. Data collection included patient demographics, procedure information, histopathological findings, and follow-up.

### Inclusion and exclusion criteria

Inclusion criteria comprised patients with histologically confirmed HGD or esophageal adenocarcinoma (EAC) based on EMR specimens. Exclusion criteria were patients with low-grade dysplasia (LGD), those undergoing endoscopic submucosal dissection (ESD) for treatment of HGD/EAC, and those without follow-up data post-EMR.

### Variables and definitions

Baseline characteristics, such as age, gender, and body mass index (BMI), were documented. During initial endoscopy, the circumferential and maximal length of BE was assessed and described according to the Prague criteria 11 . Successful endoscopic resection was defined as low risk of lymph node metastasis (i.e. submucosal invasion ≤ 500 μm, no poor differentiation, and no lymphovascular invasion) and low risk of local recurrence (i.e. tumor-free deep resection margin). As for adverse events (AEs), delayed bleeding was defined as clinical signs of bleeding with a drop in hemoglobin > 2 g/dL. Esophageal stricture was defined as a stricture that could not be passed with a regular gastroscope. Complete remission of neoplasia (CRN) was defined as complete absence of endoscopic and histological evidence of neoplasia (HGD and EAC) on follow-up. Complete remission of intestinal metaplasia (CRIM) was defined as complete absence of endoscopic evidence of BE and histological evidence of intestinal metaplasia on follow-up. Follow-up outcomes included patients who achieved successful endoscopic resection. Recurrence of neoplasia was defined as any recurrence of HGD or EAC after CRN. This recurrence of neoplasia included local recurrence and metachronous lesions after CRN, which were not differentiated.

### Outcomes

The primary outcome was the long-term recurrence rate of neoplasia after CRN, assessed by Kaplan-Meier analysis. Secondary outcomes were the rate of residual/metachronous neoplasia at the first follow-up, the rate of CRN, and the long-term rate of patients who were successfully managed by endoscopy. This reflected the current status of CRN, regardless of any prior recurrences, among all patients who underwent successful endoscopic resection.

### EMR protocol

A therapeutic gastroscope (GIF1T-160 or GIF 1T-190, Olympus Co., Tokyo, Japan) was used for the procedure. EMR was conducted using either the cap technique 12 or multiband ligation, which involves a band-and-cut method 13 14 . All specimens were sent for histopathological analysis. Patients underwent follow-up endoscopy approximately every 3 to 6 months post endoscopic treatments until CRN and CRIM were achieved. Ablation techniques including radiofrequency ablation (RFA), photodynamic therapy (PDT), and cryotherapy were performed in cases in which residual/metachronous BE was detected on follow-up. In addition, all patients received once- or twice-daily proton pump inhibitor therapy during and after endoscopic treatment.

### Histological analysis

Histological assessments were performed by at least two gastrointestinal pathologists. EMR specimens were evaluated based on several criteria, including depth of tumor infiltration, grade of differentiation, presence of lymphatic or vascular invasion, and completeness of resection at the deep (vertical) margin. Histological criteria, classification, and differentiation grading were based on the World Health Organization classification 15 .

Biopsies were evaluated for presence of dysplasia and intestinal metaplasia.

### Post‑endoscopic treatment surveillance

Follow-up was scheduled every 3 to 6 months during the period aimed at eradication of intestinal metaplasia, and then annually thereafter. Systematic biopsies were conducted in all four quadrants of the esophagus at 1-cm intervals along the initial length of the BE segment, in addition to biopsies at the gastroesophageal junction (GEJ) in accordance with the Seattle protocol 16 . In addition, targeted biopsies were taken from any visually detected abnormalities.

### Statistical analysis

Continuous variables were expressed as a mean with standard deviation (SD), or median with interquartile range (IQR) based on normality of distribution. The long-term recurrence rate (cumulative incidence of recurrence) was evaluated with a time to event analysis using Kaplan-Meier curve. Recurrence rates were presented as percentages with their 95% confidence intervals (CIs). Statistical analyses were conducted using JMP Pro 17 software (SAS Institute Inc. Cary, North Carolina, United States).

### Ethics considerations

Each patient provided written informed consent for all procedures and participation in the prospective institutional registry. The study received approval from the Research Ethics Board (REB 08–265) on December 19, 2008.

## Results

### Patient characteristics and procedure outcomes

A total of 552 consecutive patients were diagnosed with Barrett’s HGD/EAC and were treated with EMR between January 2001 and December 2023. The patient cohort was predominantly male (462 patients, 83.7%), with a mean age of 66.3 ± 11.2 years and a mean BMI of 29.0 ± 6.4 kg/m ^2^ . Median Barrett’s length based on Prague classification was circumferential 1 cm (0–4) and maximal 4 cm (2–6). Barrett’s types were 199 short-segment BE (36.1%) and 353 long-segment BE (63.9%). Mean lesion size was 3.67 ± 2.37 cm, with a mean circumferential occupancy of 47.3 ± 20.8 %. Mean procedure time was 58.1 ± 22.0 minutes. AEs included delayed bleeding in four cases (0.72%) and perforation in two cases (0.36%) ( Table 1 ).

**Table:** **Table 1** Population data and procedure outcomes.

	**N = 552**
Age, mean (SD)	66.3 (11.2)
Male gender	462 (83.7%)
BMI, mean (SD)	29.0 (6.4)
Prague C, cm, median (IQR)	1 (0–4)
Prague M, cm, median (IQR)	4 (2–6)
Barrett’s length	
SSBE	199 (36.1%)
LSBE	353 (63.9%)
Size of the lesion, cm, mean (SD)	3.67 (2.37)
Circumferential occupancy of the lesion, mean, (SD)	47.3 (20.8)
Operative time, mean (SD)	58.1 (22.0)
Adverse events	
Delayed bleeding	4 (0.72%)
Perforation	2 (0.36%)
Histology	
High-grade dysplasia	124 (22.5%)
Adenocarcinoma	428 (77.5%)
Differentiation	N = 428
Well-differentiated	293 (68.4%)
Moderately-differentiated	112 (26.1%)
Poorly-differentiated	23 (5.3%)
Invasion depth	N = 428
T1a	369 (86.2%)
T1b SM1	43 (10.0%)
T1b SM2 or deeper	16 (3.7%)
Lympho-vascular invasion	N = 428 30 (7.0%)
Vertical margin positive	N = 428 32 (7.4%)
Successful endoscopic resection	475 (86.1%)
Additional treatment for high-risk patients	
Esophagectomy	30 (5.4%)
Chemoradiotherapy	10 (1.8%)
Close Follow-up	37 (6.7%)
All values are expressed as percentages among both groups. BMI, body mass index; IQR, interquartile range; LSBE, long-segment Barrett’s esophagus; SD, standard deviation; SSBE, short-segment Barrett’s esophagus.	

### Histopathological findings and identification of successful endoscopic resection

HGD was present in 124 patients (22.5%), and esophageal adenocarcinoma (EAC) in 428 patients (77.5%). Tumor differentiation was well-differentiated in 293 cases (68.4%), moderately-differentiated in 112 (26.1%), and poorly-differentiated in 23 (5.3%). Invasion depth was T1a in 369 cases (86.2%), T1b SM1 in 43 (10.0%), and T1b SM2 or deeper in 16 (3.7%). Based on histopathological results, successful endoscopic resection was achieved in 475 patients (86.1%). For those considered as having non-curative or unsuccessful endoscopic resection (high risk for lymph node metastasis or positive deep margin), additional treatments included esophagectomy in 30 cases (5.4%), chemoradiotherapy in 10 cases (1.8%), and close follow-up in 37 cases (6.7%).

### Short-term follow-up outcomes in patients with successful endoscopic resection

**Fig. 1:**
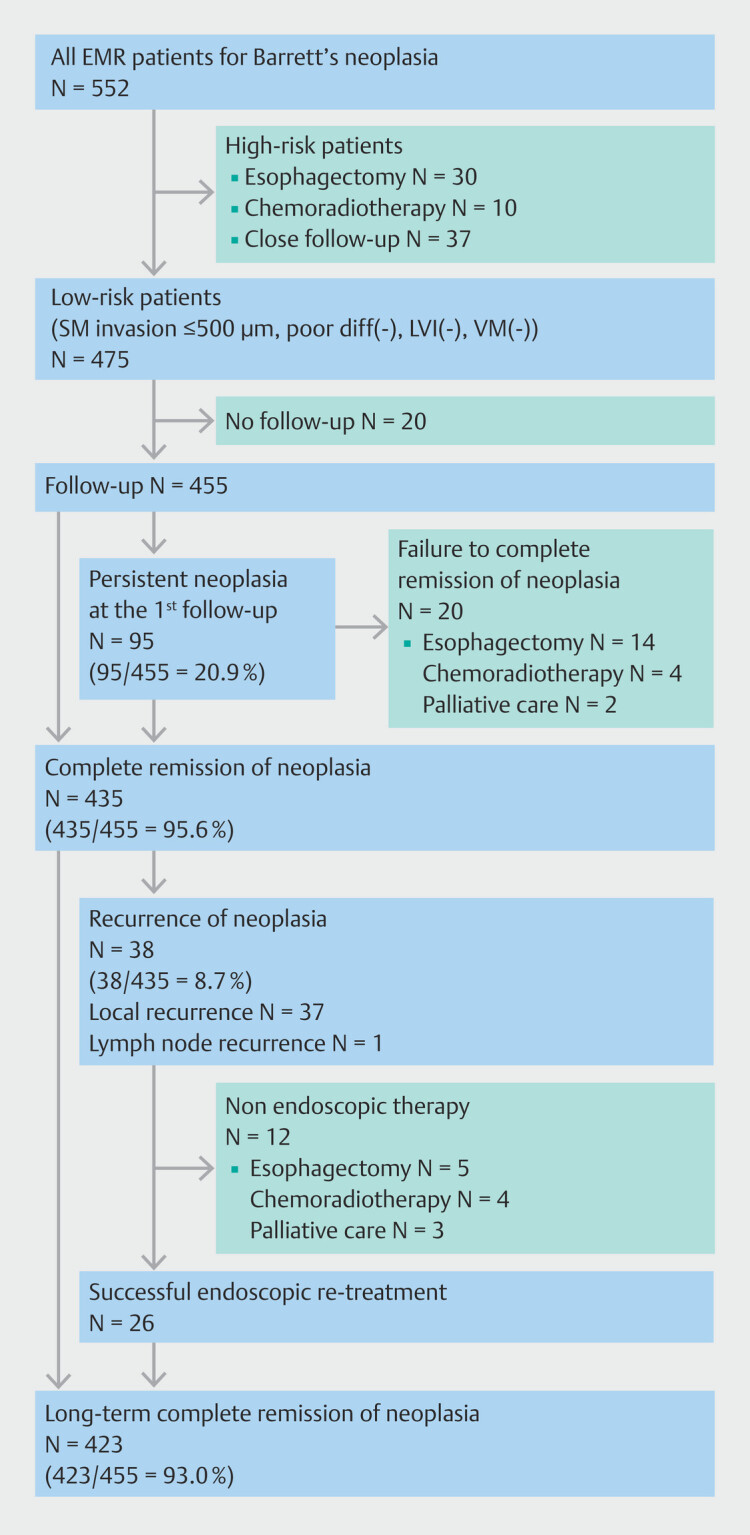
Flowchart of study patients.

Of 475 patients with successful endoscopic resection, 455 patients underwent follow-up. At initial follow-up endoscopy, residual/metachronous neoplasia was observed in 95 cases (20.9%) (95/455). After additional EMR, a total of 435 patients (95.6%) achieved CRN after undergoing a median of two EMR sessions (IQR 1–4). Twenty patients (4.4%) did not achieve CRN, and of them, 14 underwent esophagectomy, four patients received chemoradiotherapy, and two patients entered palliative care. CRIM was achieved with only EMR in 40.2% (183/455). Ablations techniques were performed following EMR in 37.6% (170/455). After undergoing a median of two ablation sessions (IQR 1–3), CRIM was achieved in an additional 23.9% of patients (109/455). In total, 64.1% (292/455) of the follow-up cohort achieved CRIM ( Fig. 1 , Table 2 ).

**Table:** **Table 2** Follow-up outcomes for successful endoscopic resection patients.

	**N = 455**
Residual neoplasia at first follow-up	95 (20.9%)
Number of EMR sessions until CRN, median (IQR)	2 (1–4)
Complete remission of neoplasia (CRN)	435 (95.6%)
Non-CRD	20 (4.4%)
Treatment for non-CRN	
Esophagectomy	14
Chemoradiotherapy	4
Palliative care	2
Stricture formation	160 (35.2%)
Number of dilations, median (IQR)	2 (1–5)
Ablation techniques performed	170 (37.6%)
RFA	148
APC	1
Cryotherapy	4
PDT	17
Number of ablation sessions until CRIM, median (IQR)	2 (1–3)
Complete remission of intestinal metaplasia (CRIM)	292 (64.1%)
CRIM after only EMR	183 (40.2%)
CRIM after EMR and ablations	109 (23.9%)
Recurrence of neoplasia after CRN	N = 435 38 (8.7%)
Local recurrence of neoplasia	N = 435 37 (8.5%)
Lymph node recurrence	N = 435 1 (0.2%)
Treatment for recurrence	
Endoscopic treatment	26
Esophagectomy	5
Chemoradiotherapy	4
Palliative care	3
Total follow-up period, months, mean (SD)	50.0 (39.3)
All values are expressed as percentages among both groups. APC, argon plasma coagulation; CRIM, complete remission of intestinal metaplasia; CRN, complete remission of neoplasia; IQR, interquartile range; PDT, photodynamic therapy; RFA, radiofrequency ablation; SD, standard deviation.	

### Long-term follow-up outcomes of patients who achieved CRN

During a mean total follow-up period of 50.0 ± 39.3 months, recurrence of neoplasia occurred in 38 of 435 patients (8.7%) who had achieved CRN. This includes local recurrence of neoplasia in 37 cases (8.5%) and lymph node recurrence in one case (0.2%). Of the 38 patients with recurrence, 26 (68.4%) were successfully treated endoscopically, but 12 patients (31.6%) were not: five underwent esophagectomy, four received chemoradiotherapy, and three entered palliative care.

Among the entire follow-up cohort of 455 patients, 93.0% (423/455) were successfully managed with endoscopic therapy. In contrast, 32 patients (7%) failed to achieve CRN or had recurrence of neoplasia leading to esophagectomy, chemoradiotherapy, or palliative care.

**Fig. 2:**
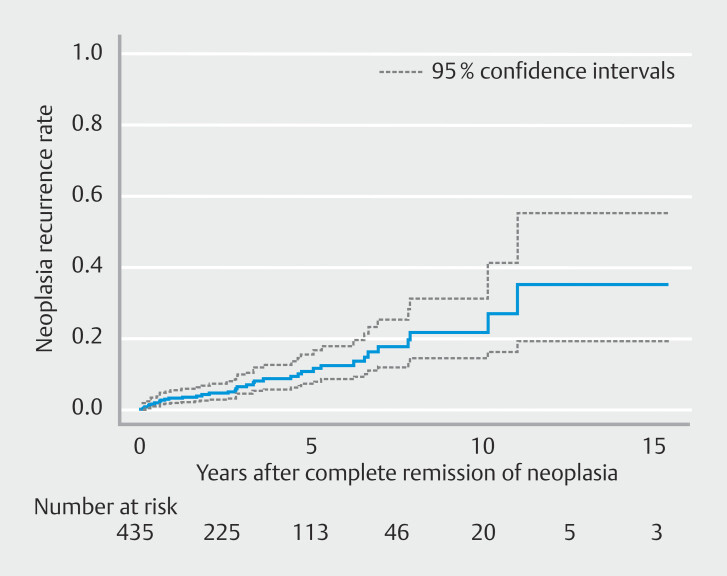
Kaplan-Meier curve of cumulative incidence of neoplasia recurrence.

The Kaplan-Meier curve representing cumulative incidence of neoplasia recurrence after CRN is shown in Fig. 2 . As a primary outcome, the 1-year recurrence rate was 3.1% (95% CI 1.4–4.7), the 3-year recurrence rate was 6.3% (95% CI 3.5–9.0), the 5-year recurrence rate was 10.5% (95% CI 6.4–14.6), the 10-year recurrence rate was 21.6% (95% CI 13.2–29.9), and the 15-year recurrence rate was 34.9% (95% CI 16.2–53.7) ( Table 3 ).

**Table:** **Table 3** Cumulative incidence of neoplasia recurrence following complete remission of neoplasia.

	**Recurrence rate**	**95% CI**
1-year	3.1%	1.4%-4.7%
3-year	6.3%	3.5%-9.0%
5-year	10.5%	6.4%-14.6%
10-year	21.6%	13.2%-29.9%
15-year	34.9%	16.2%-53.7%
CI, confidence interval.		

**Fig. 3:**
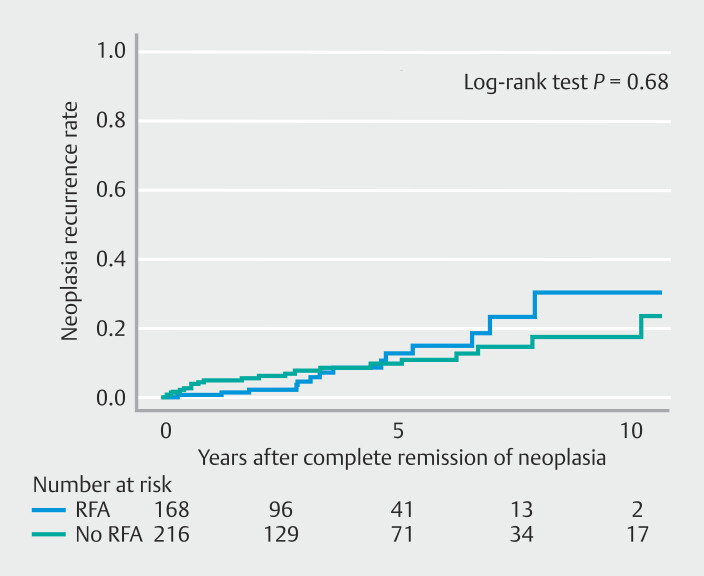
Kaplan-Meier curve of cumulative incidence of neoplasia recurrence in patients who underwent ablations vs. those who did not.

The Kapan Meier curve representing cumulative incidence of neoplasia recurrence in patients who underwent ablations vs. those who did not is shown in Fig. 3 . The 1-year recurrence rate for patients who underwent RFA was 0.6% (95% CI 0–1.8), the 3-year recurrence rate was 4.5% (95% CI 0.52–8.48), the 5-year recurrence rate was 12.7% (95% CI 4.7–20.6), and the 10-year recurrence rate was 30.2% (95% CI 12.2–48.2). The 1-year recurrence rate for patients who did not undergo RFA was 4.8% (95% CI 2.0–7.5), the 3-year recurrence rate was 7.6% (95% CI 3.7–11.4), the 5-year recurrence rate was 9.5% (95% CI 4.9–14.1), and the 10-year recurrence rate was 17.5% (95% CI 8.6–26.3). There was no statistically significant difference in recurrence rate between the two groups ( *P* = 0.68, log-rank test).

## Discussion

In this large, single-center, retrospective cohort study, we investigated long-term outcomes of EMR for Barrett's neoplasia, with a particular focus on recurrence of neoplasia. Although the overall rate of patients who were successfully managed by endoscopy was 93%, recurrence rates for neoplasia following CRN were observed to be considerable over the long term, reaching 10.5% at 5 years, 21.6% at 10 years, and 34.9% at 15 years.

EMR for BE has become one of the standard procedures for treating BE HGD and EAC. Guidelines recommend histopathological staging based on endoscopic resection of visible lesions within BE 7 8 9 . Following endoscopic resection, patients are categorized based on their risk of lymph node metastasis and local recurrence, as predicted by histopathology. For patients assessed as low-risk, endoscopic follow-up is recommended 9 . Several studies have examined long-term recurrence rates following EMR for Barrett’s neoplasia. One study reported a 6.2% recurrence rate for neoplasia over a mean follow-up of 64.8 months post-EMR for lesions including LGD to adenocarcinoma 17 . Another study showed that the recurrence rate for HGD or EAC was 14.5% over a mean follow-up period of 56.6 months post-EMR for intramucosal adenocarcinoma 3 . Our study indicated that the 5-year recurrence rate was 10.5%, the 10-year recurrence rate was 21.6%, and the 15-year recurrence rate was 34.9%. Our findings align with previous reports, but this is the first report to show long-term recurrence rates beyond 10 years.

In the current guideline, for patients with a baseline diagnosis of HGD or EAC, surveillance endoscopy is recommended at 1, 2, 3, 4, 5, 7, and 10 years after last treatment 9 . This guideline also indicates that surveillance may be discontinued after 10 years 9 . However, our findings demonstrate that recurrence rates continue to increase over time, even beyond the 10-year mark. This evidence suggests that continuing surveillance endoscopy beyond 10 years should be considered, taking into account patient age and comorbidities.

In our study of 435 patients who underwent successful endoscopic resection and completed follow-up endoscopy, 93.0% (423/455) were successfully managed with endoscopic treatments. This confirms the high efficacy of EMR in management of Barrett’s neoplasia. Previous studies reported a similar rate of successful endoscopic management of 93.8% 3 , which is consistent with our results.

In contrast, 32 patients in our study (7%) who were initially considered as having had successful endoscopic treatment ultimately required esophagectomy or chemoradiotherapy, or entered palliative care. This includes 20 patients who were initially considered successfully treated by EMR but ultimately failed to achieve CRN due to residual/metachronous neoplasia and required more invasive therapy. Repeat attempts at EMR on the same area become increasingly challenging as the neoplastic area becomes fibrotic and scarred down. Furthermore, 31.6% of patients who had neoplastic recurrence (12/38) had lesions that were not amenable to endoscopic management. Similar challenges in treating recurrent lesions are that the lesions become embedded in fibrotic areas. For band ligation or cap EMR, suction of lesions into the cap is particularly difficult when lesions are within scarred or fibrotic areas. If endoscopic treatment is not feasible, more invasive approaches such as esophagectomy, chemoradiotherapy, or palliation were often necessary, even for lesions that were not advanced cancers.

The current guideline recommends en bloc resection by ESD for lesions suspected of submucosal invasion, for malignant lesions larger than 20 mm, and for lesions in scarred or fibrotic areas 9 . In addition, recent studies have demonstrated a lower recurrence rate following ESD compared with EMR 18 19 . Most of our study data come from a period before the widespread availability of ESD in the West. However, use of ESD for appropriate lesions could potentially reduce recurrence rates and increase the success rate for treatment of recurrent lesions in fibrosis. Consequently, judicious selection of resection techniques is likely to improve the proportion of patients who can be successfully managed with endoscopy.

In this study, ablation techniques were performed in 37.6% of patients (170/455), resulting in achievement of CRIM in 64.1% (292/455). Adoption of ablation techniques for BE became common following introduction and validation of RFA 20 . The relatively low rate of ablation technique use in our study can be attributed to our cohort encompassing the period before RFA became common. CRIM was achieved by EMR alone in only 40.2% (183/455), with ablation techniques performed following EMR in 37.6% (170/455) and CRIM achieved in an additional 23.9% of patients (109/455). Interestingly, there was no statistically significant difference in recurrence rate between the groups of patients who underwent RFA compared with those who did not ( *P* = 0.68), which is potentially explained by the increased tendency to perform widefield EMR of BE prior to the RFA era. We acknowledge that our lower rate of CRIM is relevant because some studies have indicated that failure to achieve CRIM is associated with an increased risk of dysplasia recurrence 21 .

This study has several limitations. First, its retrospective design and extended follow-up period inevitably led to some patients being lost to follow-up. To account for this, we employed the Kaplan-Meier method, which accommodates censoring and provides an estimate of recurrence probability over time. This approach helps mitigate selection bias associated with loss to follow-up. The Kaplan-Meier analysis assumes that patients lost to follow-up have a similar prognosis (i.e., recurrence risk) as those who remain under observation. To assess this assumption, we conducted a comparative analysis of patient and lesion characteristics, as well as follow-up outcomes, between individuals with shorter follow-up durations (< 5 years) and those with longer follow-up durations (≥ 5 years) ( **Supplementary Table 1** ). The results demonstrated no significant differences in lesion characteristics between the two groups. Although patients with longer follow-up had longer BE segments, they also exhibited a higher rate of CRIM. Furthermore, recurrence rates were comparable between the short- and long-term follow-up groups. Given that achieving CRIM has been reported to significantly reduce recurrence risk 21 , these findings suggest that patients with longer follow-up do not have an increased risk of recurrence compared to those with shorter follow-up. Nonetheless, our study observed a high cumulative recurrence rate, with estimated rates of 21.6% at 10 years and 34.9% at 15 years. However, to more accurately assess recurrence risk, future studies with more comprehensive and complete follow-up data are warranted to minimize potential biases associated with loss to follow-up.

Second, endoscopic techniques and tools have evolved over time, particularly devices used for EMR and ablation. Third, indications for EMR have shifted over time. Prior to the advent of RFA, EMR was often utilized to completely eradicate the entire Barrett’s segment. With widespread adoption of RFA, the focus of EMR shifted to resection of visible lesions. More recently, larger lesions or those suspected to harbor more advanced pathology are preferentially selected for treatment with ESD, whereas EMR has often been reserved for smaller, less invasive lesions. This shift in indications is likely to have influenced outcomes in our study. Fourth, RFA is currently performed following EMR with the aim of achieving CRIM as standard of care. Therefore, reduction in recurrence rates due to RFA also affects the recurrence rates following EMR. However, the data in our study reflect real-world clinical situations, which underscores the importance of reporting recurrence data that accurately represent actual clinical practice. Finally, it is possible that AEs may not have been fully captured within our cohort. This is because patients who experienced post-procedure AEs may have sought care at other medical centers, which we may have failed to capture. This may have resulted in underestimation of the AEs in this study. However, given that AE rates were not an outcome of interest, no additional methodology was employed to ensure comprehensive documentation of all AEs.

## Conclusions

In summary, in this large, single-center, retrospective cohort study, long-term outcomes following EMR for Barrett's neoplasia were investigated. Although the overall rate of patients who were successfully managed by endoscopy was excellent, the recurrence rate over time was notably high. These data imply that surveillance endoscopy beyond 10-year follow-up should be considered.

## Supplementary Material

Supplementary Material Zusatzmaterial
