# Supplementary material for: Endoscopic mucosal resection for Barrett’s neoplasia: Long-term outcomes from the largest Canadian single-center experience
Source: Endosc Int Open. 2025 Jun 17;13:a26028961. doi: 10.1055/a-2602-8961 (PMC12223934; doi:10.1055/a-2602-8961)
Supplement: Supplementary file 1 — Supplementary Material [file 10-1055-a-2602-8961_26029771.pdf]

Supplementary Table 1 Patient characteristics and treatment outcomes based on follow-up duration after CRN: Comparison of patients followed < 5 years vs. ≥ 5 years

	**< 5 years of follow-up N = 323**	**≥ 5 years of follow-up N = 112**	***P* value**
Age, mean (SD)	66.3 (11.0)	64.1 (11.0)	0.068
Male gender	262 (81.1%)	101 (90.2%)	0.13
Prague C, cm, median (IQR)	1 (0-4)	2 (0-5)	**0.088**
Prague M, cm, median (IQR)	3 (2-6)	4 (2-6)	**0.046**
Size of the lesion, cm, mean (SD)	3.4 (2.2)	4.0 (2.4)	0.11
Histology			0.92
High-grade dysplasia	91 (28.17%)	31 (27.68%)	
Adenocarcinoma	232 (71.83%)	81 (72.32%)	
Differentiation	N = 232	N = 81	0.12
Well-differentiated	173 (74.57%)	68 (83.95%)	
Moderately-differentiated	59 (25.43%)	13 (16.05%)	
Invasion depth	N = 232	N = 81	0.38
T1a	220 (68.11%)	80 (71.43%)	
T1b SM1	12 (3.72%)	1 (0.89%)	
Lymphovascular invasion	1 (0.43%)	0 (0.00%)	0.55
Vertical margin positive	0 (0%)	0 (0%)	1.00
Ablation performed	128 (39.63%)	41 (36.61%)	0.65
CRN	323 (100.00%)	112 (100.00%)	1.00
CRIM	193 (59.75%)	99 (88.39%)	**< 0.001**
Local recurrence	27 (8.36%)	10 (8.93%)	0.85

CRN, complete remission of neoplasia; CRIM, complete remission of intestinal metaplasia; IQR, interquartile range; SD, standard deviation.

Endosc Int Open | DOI 10.1055/a-2602-8961 | © The Author(s).
